# Biocompatible Black Phosphorus Nanosheets-Antimicrobial Peptide Nanocomposites for Enhanced Anti-Infection Therapy

**DOI:** 10.3390/molecules30040872

**Published:** 2025-02-14

**Authors:** Shuo Liu, Zhishang Shi, Lin Teng, Junlian Nie, Libing Zhang

**Affiliations:** 1 School of Energy and Chemical Engineering, Tianjin Renai College, Tianjin 301636, China; teng_lin@tju.edu.cn (L.T.); niejl24@163.com (J.N.); 2 College of Life Sciences, Nankai University, Tianjin 300071, China; shizs@mail.nankai.edu.cn; 3 Tianjin Key Laboratory of Molecular Optoelectronic Sciences, Department of Chemistry, School of Science, Tianjin University, Tianjin 300072, China; libing.zhang@tju.edu.cn

**Keywords:** black phosphorus, antimicrobial peptide, antibacterial, wound healing

## Abstract

Bacterial infections are one of the major problems affecting human health, which is exacerbated by increasing antibiotic resistance. Antimicrobial peptides (AMPs) are an ideal alternative to antibiotics, but their instability and toxicity to mammalian cells need to be addressed. Here, black phosphorus nanosheets (BPs) were successfully decorated with melittin (Mel), a kind of AMP, through electrostatic interaction. The size impacts of BPs on the antibacterial ability and biocompatibility of BPs/Mel nanocomposites were studied systematically. Results showed that the nanocomposites made from middle-sized BPs (BPs/Mel-7) have strong antibacterial ability as well as good biocompatibility. Moreover, BPs/Mel-7 could accelerate skin wound healing infected by *Staphylococcus aureus*. This study provides a facile strategy to expand the application of AMPs.

## 1. Introduction

The rapidly increasing antibiotic resistance in pathogens caused by antibiotic abuse is a severe problem for global healthcare and the economy [1,2]. Alternatives with powerful antimicrobial ability and minimal antimicrobial resistance are urgently needed. As a kind of promising candidate, natural and artificial antimicrobial peptides (AMPs) have attracted great attention in research and clinical to overcome antibiotic resistance due to their broad biological activities, including but not limited to antibacterial, antiviral, antifungal, and even antitumor properties [3,4,5]. However, drawbacks such as easy degradation by proteases as well as potential toxicity toward eukaryotic cells (especially hemolysis) hinder their wide therapeutic application [6].

With the advancement of nanotechnology, nanomaterials-based multifunctional antimicrobials have also been developed [7]. On the one hand, functional nanomaterials may exhibit intrinsic or light-assisted antimicrobial properties with the mechanisms of physical damage to cell membranes, chemical damage to cellular components, and suppression of bacterial metabolism [8,9,10]. On the other hand, elaborately engineered nanomaterials can act as delivery platforms for antibacterial agents (e.g., AMPs) to protect them from degradation, reduce side effects, and regulate their release, which significantly improves the antibacterial effects [11,12]. Despite great progress in antimicrobial nanomaterials, in vivo applications remain limited due to their cytotoxic effects. For instance, silver nanoparticles with broad-spectrum antibacterial ability also showed toxicity to human cells [13]. Consequently, the search for highly biocompatible nanomaterials that are appropriate to deliver antibacterial agents or intrinsic antimicrobials continues to be an ongoing effort.

As a novel number of 2D nanomaterials, black phosphorus nanosheets (BPs) have attracted great attention for potential biomedical application because of their unique properties, such as high surface area and drug loading capacity, biocompatibility, and most importantly, biodegradability as BPs can be degraded to nontoxic PO_4_^3−^ [14,15,16]. Particularly, several recent studies reveal that BPs can be regarded as promising candidates to combat bacteria [17,18] with the mechanisms of physical damage of cell membrane (lipid extraction, nano-knife effects), photothermal effects, generation of reactive oxygen species (ROS), and loading of other antibacterial agents to construct synergistic antibacterial platforms.

Inspired by the characteristics of BPs and AMPs, using a typical AMP, i.e., Melittin (Mel), we plan to prepare a nanocomposite consisting of BPs and Mel (BPs/Mel) to obtain potent antibacterial ability and minimize the cytotoxic of Mel. As AMPs are positively charged, it is speculated that AMPs can easily bind to the negatively charged BPs through electrostatic interactions. It is reported that different size of BPs obtained by gradient centrifugation can strongly affect their antimicrobial mechanisms, e.g., nano-knife effects for bigger-sized BPs (>400 nm) [19] and reactive oxygen species (ROS) generation for smaller-sized BPs (less than 100 nm) [20]. In this study, BPs ranging in size from ~70 to ~430 nm were used to prepare BPs/Mel, and their antibacterial ability and cytotoxicity as a function of size were investigated. We identified that the nanocomposites made from middle-sized BPs maintain strong antibacterial ability and show the highest cytocompatibility.

## 2. Results and Discussion

### 2.1. Characterization of BPs and BPs/Mel Nanocomposites

**Figure 1:**
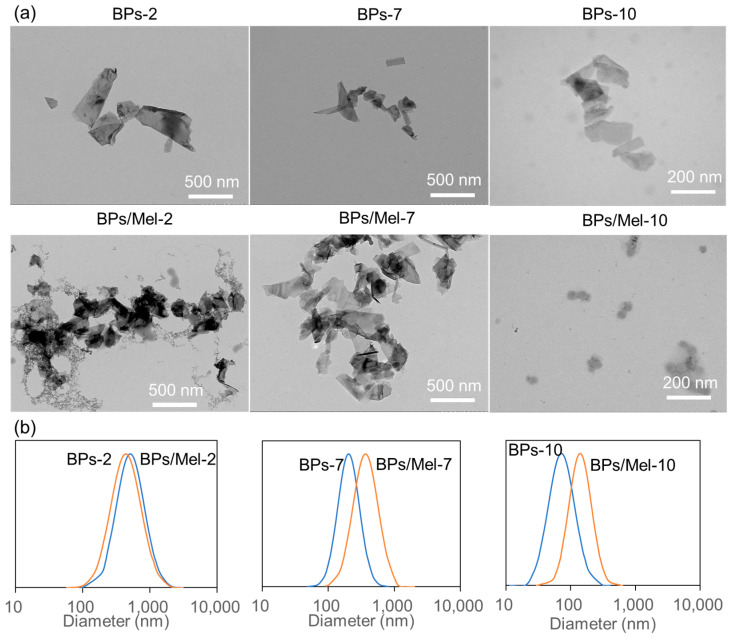
(**a**) TEM images of BPs and BPs/Mel. (**b**) DLS graphs of BPs and BPs/Mel.

Monodispersed BPs of different sizes were prepared by ultrasonication-assisted liquid exfoliation of bulk BP and followed by controlled gradient centrifugation [21]. Three centrifugation rate ranges, 1000 to 2000 rounds per minute (rpm), 4000 to 7000 rpm, and 10,000 to 14,800 rpm, were used to get the corresponding BPs, each of which was marked as BPs-2, BPs-7, and BPs-10, respectively. Figure 1 and Table S1 depict the transmission electron microscope (TEM) and dynamic light scattering (DLS) characterization of three BPs. As shown in Figure 1, all BPs exhibit 2D morphology, and the average lateral size decreased with increasing centrifugation speed. DLS results were consistent with TEM results, as the average diameter of BPs-2, BPs-7, and BPs-10 were 430.6, 201.7, and 71.5 nm, respectively (Table S1 & Figure 1b). The high surface-to-volume ratio and puckered lattice configuration of BPs make it an excellent carrier for small molecules and biomolecules. In particular, the negatively charged BPs can load positively charged drugs through electrostatic interaction with high loading capacity [22,23]. BPs were modified with positively charged AMPs, Mel, via electrostatic interaction, taking advantage of the negative charge and facile surface functionalization. Mel, a major component of honeybee (*Apis mellifera*) venom, has great therapeutic values in anti-cancer, anti-bacterial, anti-fungal, and other biomedical fields [24]. By mixing Mel with different-sized BPs in PBS at pH 7.4, the BP surface was decorated with Mel. Since the zeta potential of Mel is 16 mV at neutral pH, modification of Mel is expected to shift the BPs potential from negative (BPs) to positive (BPs/Mel). As shown in Table S1, all zeta potential of the three sized BPs/Mel shifted to positive values. As shown in Figure 1, the BPs/Mel nanocomposites exhibited 2D morphology as well; the lateral size is similar to corresponding BPs but tends to form aggregation. DLS results indicated that the average diameter of BPs/Mel-2, BPs/Mel-7, and BPs/Mel-10 were 504.8, 363.19, and 143.6 nm, respectively (Table S1 & Figure 1b). The polydispersity index (PDI) in Table S1 showed that the size distribution of BPs/Mel-2 and BPs/Mel-10 became narrower compared with BPs-2 and BPs-10, respectively, while the size distribution of BPs/Mel-7 was broader than BPs-7. Of course, as the size control of the black phosphorus nanosheet depends on the centrifugal speed, the size change range of the nanosheet obtained at the selected speed is not very different [21]. Moreover, it was found that ~1.5 μg of Mel adsorbed per μg of BPs (Figure S1).

### 2.2. Antibacterial Activity of BPs/Mel

Prior to antibacterial application, the cytotoxicity of BPs/Mels in vitro was tested by CCK-8 assay [25]. As present in Figure S2a, strong cytotoxicity in vitro was observed by incubating Hela cells with 50 μg/mL of Mel, further demonstrating that Mel has toxicity toward eukaryotic cells [26,27]. On the other hand, BPs are biocompatible, and BPs with different sizes at the same concentration exhibit no obvious cytotoxicity. Interestingly, Hela cells incubated with 50 μg/mL of BPs/Mel-2 and BPs/Mel-7 also showed no cytotoxicity; however, the smallest-sized BPs/Mel-10 showed slight toxicity to cells, as the cell viability was below 70%. As the nanocomposites contain approximately 60% of Mel by weight, it is clear that the nanocomposites possess higher biocompatibility than Mel. However, by decreasing the dosage of Mel to 25 μg/mL, the cell viability was still below 30% (Figure S2b), demonstrating the incorporation of BPs decreases the cytotoxicity of Mel significantly.

Considering the potential cytotoxicity of BPs/Mel-10, we then evaluated the antibacterial ability of BPs/Mel-2 and BPs/Mel-7 and calculated the 50% inhibitory concentration (IC_50_) against *E. coli* and *S. aureus*. As shown in Table 1, both BPs-2 and BPs-7 exhibited no antibacterial ability under our tested concentration, which is acceptable as previously reported [28,29]. Mel showed good antibacterial ability against both strains, and the IC_50_ value toward *E. coli* was a little higher, which was consistent with our previously reported results [26]. With the introduction of Mel, the antibacterial ability of BPs/Mel was much higher than BPs, and the IC_50_ value of BPs/Mel-7 was much higher than BPs/Mel-2, which was mainly attributed to the size effect of black phosphorus nanosheets, the smaller the size, the stronger the antibacterial ability [19,28]. Moreover, the IC_50_ value of BPs/Mel-7 was similar to Mel, considering the cytotoxicity of Mel, BPs/Mel-7 may be suitable for antibacterial application in vivo.

**Figure 2:**
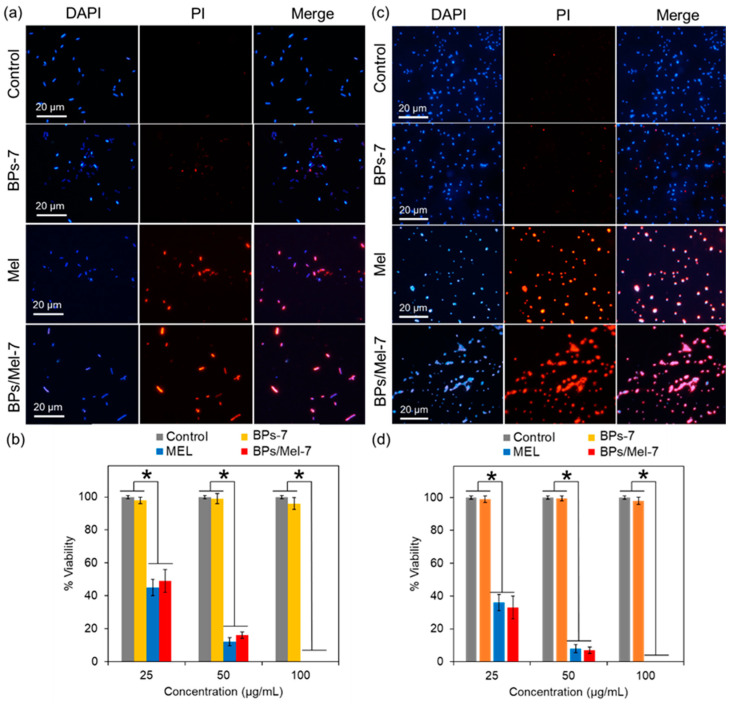
Antibacterial ability of BPs-7, Mel, and BPs/Mel-7. Confocal fluorescent images of live and dead *E. coli* (**a**) and *S. aureus* (**c**), the concentration of different agents is 25 mg/L, DAPI, 4′,6-diamidino-2-phenylindole. (**b**,**d**) Bacterial viability statistics based on confocal results. The asterisks (*) indicate significant differences between the groups (*p* < 0.05).

The antibacterial ability of Mel/BPs-7 was then studied systematically. Figure 2a,c is the fluorescence confocal images of bacteria treated with 50 μg/mL agents. Propidium iodide (PI) specifically stained dead bacteria and showed red fluorescence under 535 nm excitation. It can be seen from the figure that the number of bacteria with red fluorescence significantly increased when Mel and BPs/Mel-7 were added, and most of the blue fluorescence overlaps (Merge, Figure 2), indicating that most of the bacteria have died. When BPs-7 was incubated alone, the number of bacteria with red fluorescence was very small. Figure 2b,d showed the antibacterial effect according to the fluorescence confocal results. It can be seen that the antibacterial effect of BPs-7 alone is not obvious, while Mel and BPs/Mel-7 have obvious antibacterial ability. At a concentration of 50 μg/mL, more than 80% of cells were dead, and almost all the bacteria died when increasing the concentration to 100 μg/mL.

**Figure 3:**
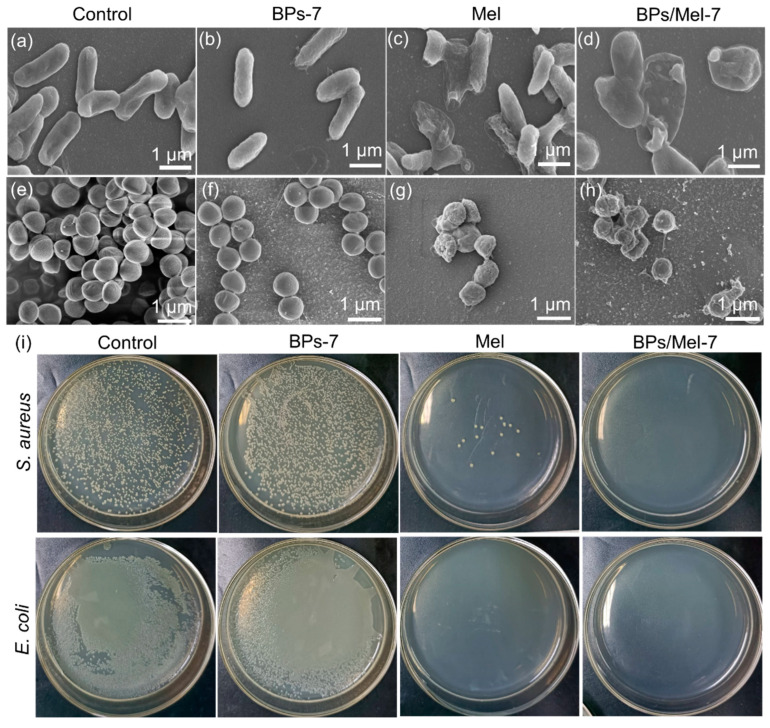
SEM morphology of *E. coli* (**a**–**d**) and *S. aureus* (**e**–**h**). (**i**) Photographs of bacterial LB agar plates.

The morphologies of *E. coli* and *S. aureus* were also observed by scanning electron microscope (SEM) after the incubation of different agents. As shown in Figure 3a–d, *E. coli* in the control group had a complete structure and smooth surface, while the morphology of *E. coli* incubated with BPs-7 did not change. After incubation with Mel, the bacterial surface folds were obvious, and some bacterial structures were destroyed. The surface folds of *E. coli* after incubation by BPs/Mel-7 were not as obvious as those in the Mel-treated group, but some spheroids of hundreds of nanometers appeared, indicating that the antibacterial mechanism of BPs/Mel-7 may be different from that of Mel. By combining the results of confocal fluorescence and scanning electron microscopy, it can be found that Mel and BPs/Mel-7 can destroy the cell membrane of *E. coli*, resulting in cytoplasmic leakage and bacterial death. There were also significant differences in the morphology of *S. aureus* after incubation of different agents. As shown in Figure 3e–h, *S. aureus*, after the incubation of Mel and BPs/Mel-7, was obviously destroyed, further proving that BPs/Mel-7 has a good antibacterial effect. The antibacterial activity of BPs-7, Mel, and BPs/Mel-7 against *E. coli* and *S. aureus* was also evaluated by counting live cells on Luria-Bertani (LB) agar plates. The bacterial cells were incubated with different agents at the same concentration (50 μg/mL) for 1 h, and then plated on LB agar plates for 24 h. As shown in Figure 3i, both Mel and BPs/Mel-7 exhibited strong antibacterial ability against *E. coli* and *S. aureus*.

**Figure 4:**
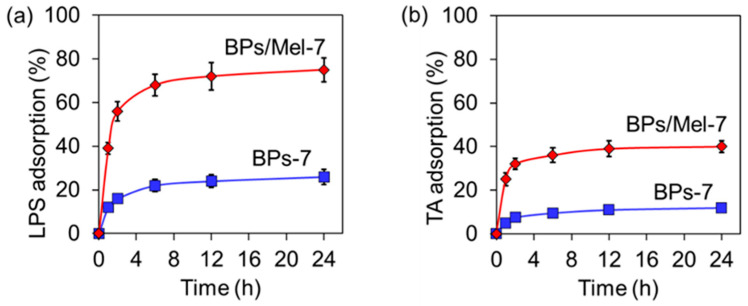
Adsorption capacity of BPs-7 and BPs/Mel-7 towards LPS (**a**) and TA (**b**).

Since BPs/Mel-7 may destroy the cell membrane and cause cell death, we speculated that BPs/Mel-7 could interact with the biomacromolecules on the cell surface. To confirm this, we chose lipopolysaccharide (LPS) [30], a typical component on the outer membrane of Gram-negative bacteria, and teichoic acid (TA) [31], a special component of the cell wall of Gram-positive bacteria, and tested the adsorption ability of BPs-7 and BPs/Mel-7 to these biomacromolecules. As shown in Figure 4a,b, both BPs-7 and BPs/Mel-7 could adsorb LPS and TA with time, and the adsorption equilibrium was reached after about 8 h. Noteworthy, the adsorption capacity of BPs/Mel-7 for the two biomacromolecules is significantly higher than that of BPs-7, suggesting that BPs/Mel-7 can adsorb bacteria more effectively, causing membrane damage [32].

### 2.3. In Vivo Antibacterial Activity of BPs/Mel-7

**Figure 5:**
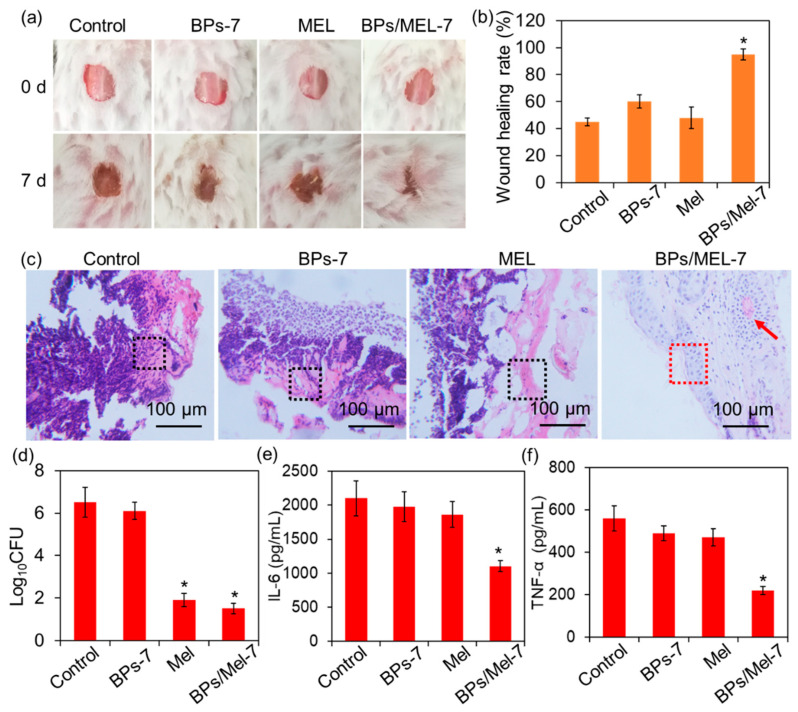
In vivo antibacterial activity of BPs/Mel against *S. aureus*. (**a**) Images of mouse wounds on day 0 and day 7. (**b**) Wound healing rate on day 7. (**c**) Histopathological images of the wound tissue on day 7. (**d**) Bacterial burden in wound tissue on day 7. (**e**) IL-6 expression in the wound. (**f**) TNF-α expression in the wound. The asterisks indicate a significant difference between the groups (*p* < 0.05).

Based on the antibacterial ability and good biocompatibility of BPs/Mel-7, the in vivo antibacterial activity was further investigated with a mouse wound infection model [33]. The wounds of the mouse were infected by *S. aureus* and then treated with PBS (Control), BPs-7, Mel, and BPs/Mel-7, respectively. After 7 days, the wound healing of mice was shown in Figure 5a. It is clear that the wound treated by BPs/Mel-7 has nearly healed, and the healing rate is about 90% (Figure 5b). Though Mel exhibited strong antibacterial activity, the wound healing rate of the Mel-treated group was not as good as BPs/Mel-7. Hematoxylin and Eosin (H&E) staining was performed on longitudinally sectioned skin tissue collected from the wound site to analyze the healing process (Figure 5c). The results showed that on day 7, significant infiltration of inflammatory cells (black dashed border) was observed in the Control, BPs-7, and Mel groups. In contrast, the BPs/Mel-7 treatment group exhibited a notable reduction in inflammatory cells, with only a minimal presence remaining. Further analysis of epidermal integrity revealed that the Control, BPs-7, and Mel groups displayed severe skin tissue damage with an incomplete epidermal structure. However, in the BPs/Mel-7 treatment group, a well-formed epidermis (red dashed border) was observed, indicating effective epidermal regeneration. Moreover, newly formed blood vessels were identified in this group (highlighted by red arrows), suggesting enhanced tissue repair following BPs/Mel-7 treatment. We also compared the bacterial load of the wounds using colony-forming unit (CFU) assays and found that the bacterial content of the BPs/Mel-7 treated group was similar to that of the Mel group, which was much lower than other groups (Figure 5d).

Inflammatory response, especially the release of inflammatory cytokines, plays a critical role in wound healing. During wound healing, the overexpression of proinflammatory factors (such as Interleukin-6, IL-6, and Tumor necrosis factor-α, TNF-α) may prevent the transition from the inflammatory phase to the proliferative phase and ultimately prolong the healing time [34,35]. On day 7, the wound tissues were sampled and homogenized; after centrifugation, the IL-6 and TNF-α levels of in supernatants were tested using ELISA kits. As shown in Figure 5e,f, the expression of IL-6 and TNF-α in the BPs/Mel-7 treated group was much lower than in other groups, demonstrating the paramount anti-inflammatory activity of BPs/Mel-7.

## 3. Materials and Methods

### 3.1. Materials

Black phosphorous (BP) crystal powder was purchased from 3Alab Chemical Technology Co., Ltd. (Shanghai, China), cell counting kit-8 (CCK-8) was purchased from Beijing Solarbio Science & Technology Co., Ltd., (Beijing, China) and N-methyl-2-pyrrolidone (NMP) was purchased from Xiensiopude Technology Co., Ltd. (Tianjin, China). All the other chemical reagents we used were analytical reagent grade without any further purification. Ultrapure water (18.2 MΩ·cm, 25 °C) was used to prepare the solutions. Melittin (Mel) was obtained from ChinaPeptides, Shanghai, China.

The *E. coli* and *S. aureus* strains were isolated from the clinic and stored in the Laboratory of Modern Mycology, Nankai University, Tianjin.

### 3.2. Preparation of BP Nanosheets (BPs) and BPs/Mel with Different Sizes

BPs with different sizes were obtained by using a solvent exfoliation technique with the assistance of gradient centrifugation. Briefly, 20 mg of BP crystal powder was added to 20 mL of NMP in a 50 mL centrifuge tube. The mixture was sonicated for 10 h using probe sonication at the power of 500 W in sealed form. The ultrasound probe worked for 2.5 s with an interval of 2 s. The temperature of the sample solution was kept below 20 °C by an ice water bath. The resulting dispersion was centrifuged for 20 min at 1000 rpm to remove multilayered BPs. Three centrifugation rate ranges, 1000 to 2000 rounds per minute (rpm), 4000 to 7000 rpm, and 10,000 to 14,800 rpm, were used to get the corresponding BPs, each of which was marked as BPs-2, BPs-7, and BPs-10, respectively. The time of centrifugation is 20 min. The sediments obtained by the three centrifugations were re-dispersed in water for further use.

For the preparation of BPs/Mel, different-sized BPs (50 μg/mL) were dispersed in PBS (pH = 7.4, 10 mM) and mixed with Mel (1 mg/mL). After deoxygenation, the mixture was ultrasounded for 30 min and shaken in the dark for 12 h. Then the mixture was centrifuged (12,000 rpm, 15 min). The supernatant was collected to test the concentration of unreacted Mel. The precipitate was washed with water 3 times to obtain BPs/Mel with different sizes.

### 3.3. Characterization

The morphology and thickness of BP nanosheets were characterized by transmission electron microscopy (TEM; FEI, Tecnai G2 F20, Hillsboro, OR, USA). Dynamic light scattering (DLS) and zeta potential were measured using a Malvern Zetasizer Nano-ZS instrument (Malvern, UK). The morphology of bacteria was characterized by a scanning electron microscope (SEM, TESCAN MIR4, Brno, Czech Republic). The UV absorption of Mel was tested by a UV-Vis spectrophotometer (U-3900, HITACHI, Tokyo, Japan).

### 3.4. Quantitative Method for Loading Amount of Mel on BPs

To ensure the loading capacity of BPs, we used an excess amount of Mel to prepare the BPs/Mel nanocomposites. UV absorption of Mel was used to quantify the amount of Mel adsorbed on BPs based on the method from [36]. The absorption spectra of increasing concentrations of Mel (in PBS buffer, 10 mM, pH = 7.4) (Figure S1a) were obtained, and the absorption at 282 nm was used to generate a calibration curve. After the reaction of BPs and Mel, the mixture is centrifuged, and the amount of free Mel in the supernatant was calculated by its absorbance at 282 nm and the calibration’s linear fit i (Figure S1b). Then, the loading amounts of Mel on BPs can be estimated.

### 3.5. Bacterial Culture

*E. coli* and *S. aureus*, the representative Gram-negative and positive bacterium, respectively, were used for the antibacterial test. Liquid Luria–Bertani (LB) medium (5 g/L yeast extract, 10 g/L tryptone, and 10 g/L NaCl) was used to cultivate the bacteria.

Typically, a single colony of bacteria was incubated in an LB liquid medium overnight at 37 °C with constant shaking. The concentration of bacteria suspension was diluted to OD_600_ (optical density at 600 nm) = 0.1 for further use.

### 3.6. Growth Inhibition Assay

100 μL of bacteria in liquid LB medium were cultured in a 96-well plate (OD_600_ = 0.01) and treated with different amounts of BPs, Mel, and BPs/Mel, with the final volume of each well being 200 μL. The bacteria were then cultured at 37 °C for 24 h. The cells in each plate were counted using a hemocytometer, and then the half inhibitory concentration (IC_50_) value was calculated.

### 3.7. Cell Viability Test

Hela cells were cultured in DMEM medium including 10% (*v*/*v*) fetal bovine serum, 100 U mL^−1^ penicillin, and 100 U mL^−1^ streptomycin in a humidified atmosphere of 5% CO_2_ at 37 °C. The cells were seeded in a 96-well plate and incubated for 24 h. Then 50 μg/mL of Mel, BPs, and BPs/Mel were added into the cell cultures, respectively, and the cells were incubated for another 24 h. Cell viability was detected by the CCK-8 assay kits (Solarbio, Beijing, China).

### 3.8. In Vitro Antimicrobial Effect of BPs/Mel

Single colonies of activated *E. coli* or *S. aureus* were selected from an LB plate, inoculated into an LB liquid medium, and cultured in a shaking bed at 37 °C overnight. The bacteria liquid was transferred to a fresh LB liquid medium, and the initial concentration was adjusted to OD_600_ = 0.1. The suspensions were divided into groups, and each group was added with different doses of BPs-7, Mel, or BPs/Mel-7. Continue shaking for 6 h. The bacteria were collected by centrifuge, resuspended in PBS, and stained by DAPI and PI (5 mg/L) for 5 min, respectively. The fluorescence of bacteria was observed by a confocal microscope. Count the number of red cells and blue cells in each field of view, and calculate the cell survival rate: survival rate (%) = (number of blue cells − number of red cells)/number of blue cells × 100%.

For SEM observation, 1 mL of strains in LB liquid medium (OD_600_ = 1) was mixed with PBS, BPs-7 (50 μg/mL), Mel (50 μg/mL), or BPs/Mel-7 (50 μg/mL), respectively. The mixture was then plated on a piece of cover glass, and the cells were fixed by 3% formaldehyde for 2 h, dehydrated by ethanol solutions (30, 50, 70, 90, and 100% of volume fraction, respectively), dried using a freeze drier, and observed using an SEM.

### 3.9. Adsorption Assay

BPs-7 and BPs/Mel-7 were mixed with LPS from *Escherichia coli* (O55:B5) (500 μg/mL, Sigma-Aldrich, Darmstadt, German) and TA from *Staphylococcus aureus* (500 μg/mL, Sigma-Aldrich), respectively. All the suspensions were shaken at 37 °C (180 rpm) and sampled at 4, 8, 12, 16, 20, and 24 h. The supernatant of the sample was used to determine the LPS and TA contents using the liquid chromatograph mass spectrometer (LC-MS) system (LCMS-2020, Shimadzu, Japan). The adsorbed mass at each equilibrium concentration was also calculated.

### 3.10. In Vivo Wound Healing Test

The wound healing capacity in vivo was tested using a mouse skin-infection model. The animal experiments were approved by the Animal Care and Use Committee at Nankai University (Approval number 2023-SYDWLL-000367). Cut a skin wound with a diameter of about 0.5 cm on the back of the mouse using surgical scissors, and then apply 100 μL of *S. aureus* suspension (OD_600_ = 0.5) to the wound. Divide the mice into 4 groups, and the wounds were treated with 200 μL of PBS, BPs-7 (50 μg/mL), Mel (50 μg/mL), and BPs/Mel-7 (50 μg/mL), respectively. The wounds were observed for 7 days. At day 7, the wounds with surrounding tissues were collected and homogenized in PBS, and the number of *S. aureus* was tested using CFU assays in solid LB plates. The levels of IL-6 and TNF-α in supernatants were tested using the corresponding ELISA kits based on the manufacturer’s instructions (Shanghai Jianglai Biotechnology Co., Ltd., China, Shanghai, China). Another part of the collected wound tissues was fixed with 4% formaldehyde, embedded in paraffin, and stained by hematoxylin and eosin (H&E) for histopathological observation.

### 3.11. Statistical Analysis

Each experiment was performed in triplicate. The data were described as mean ± standard deviation (SD). All statistical analyses were performed using the ANOVA test (*p* < 0.05) using the SPSS software (Version 22, IBM, Armonk, NY, USA).

## 4. Conclusions

In conclusion, this study developed a black phosphorus nanosheet and antimicrobial peptide nanocomposite, which is mainly through electrostatic interaction. The antibacterial ability and cytotoxicity of the nanocomposite are dependent on the size of BPs, and the nanocomposites made from middle-sized BPs (BPs/Mel-7) exhibit both strong antibacterial ability and the highest cytocompatibility. Moreover, BPs/Mel-7 showed excellent antibacterial performance in the skin wounds infected by *S. aureus* and promoted wound healing significantly. Owing to the strong antibacterial activity and good biocompatibility, BPs/Mel-7 is expected to be a new type of AMP-based nano platform for antibacterial therapy.

## Figures and Tables

**Table 1:** IC_50_ values of BPs, BPs/Mel, and Mel to the pathogenic strains.

IC_50_ (μg/mL)	BPs-2	BPs/Mel-2	BPs-7	BPs/Mel-7	Mel
*E. coli*	>200	62.1 ± 2.1	>200	19.1 ± 2.8	16.5 ± 2.2
*S. aureus*	>200	53.2 ± 1.5	>200	12.6 ± 1.3	11.8 ± 1.6

## Data Availability

Data may be available to the corresponding author, L.S., upon request.

## Supplementary Materials

The following supporting information can be downloaded at: https://www.mdpi.com/article/10.3390/molecules30040872/s1, Figure S1: Calibration of Mel concentration in solution. (a) UV-Vis spectra of increasing concentrations of Mel in 10 mM PBS buffer (pH 7.4). (b) The calibration curve was obtained by taking the absorbance at 282 nm in (a) and plotting them against the known concentrations of Mel. The curve was fitted with a linear function.; Figure S2: Cell cytotoxicity of Hela cells treated with Mel, BPs, and BPs/Mel. (a) Cell viability of Hela cells treated with 50 μg/mL Mel, BPs, and BPs/Mel. (b) Cell viability of Hela cells treated with Mel; Table S1: Diameter and zeta potential of BPs and BPs/Mel.

## References

[1] Smith W.P.J., Wucher B.R., Nadell C.D., Foster K.R. (2023). Bacterial defences: Mechanisms, evolution and antimicrobial resistance. Nat. Rev. Microbiol..

[2] Pai L., Patil S., Liu S., Wen F. (2023). A growing battlefield in the war against biofilm-induced antimicrobial resistance: Insights from reviews on antibiotic resistance. Front. Cell. Infect. Microbiol..

[3] Xia J., Ge C., Yao H. (2024). Antimicrobial peptides: An alternative to antibiotic for mitigating the risks of Antibiotic resistance in aquaculture. Environ. Res..

[4] Li G., Lai Z., Shan A. (2023). Advances of antimicrobial peptide-based biomaterials for the treatment of bacterial infections. Adv. Sci..

[5] Lyu Z., Yang P., Lei J., Zhao J. (2023). Biological function of antimicrobial peptides on suppressing pathogens and improving host immunity. Antibiotics.

[6] Mazurkiewicz-Pisarek A., Baran J., Ciach T. (2023). Antimicrobial peptides: Challenging journey to the pharmaceutical, biomedical, and cosmeceutical use. Int. J. Mol. Sci..

[7] Xie M., Gao M., Yun Y., Malmsten M., Rotello V.M., Zboril R., Akhavan O., Kraskouski A., Amalraj J., Cai X. (2023). Antibacterial nanomaterials: Mechanisms, impacts on antimicrobial resistance and design principles. Angew. Inter. Ed. Chem..

[8] Zhou L., Deng Y., Ren Y., Poon H.L., Chu W.Y., Wang H., Chan Y.K. (2024). Antibiotics-free nanomaterials against bacterial keratitis: Eliminating infections with reactive oxygen species (ROS). Chem. Eng. J..

[9] Gao B., Ye Q., Ding Y., Wu Y., Zhao X., Deng M., Zhang J., Chen M., Zhang Y., Wei X. (2024). Metal-based nanomaterials with enzyme-like characteristics for bacterial rapid detection and control. Coordin. Chem. Rev..

[10] Manivasagan P., Thambi T., Joe A., Han H., Seo S., Jeon Y., Conde J., Jang E. (2024). Progress in nanomaterial-based synergistic photothermal-enhanced chemodynamic therapy in combating bacterial infections. Prog. Mater. Sci..

[11] Imperlini E., Massaro F., Buonocore F. (2023). Antimicrobial peptides against bacterial pathogens: Innovative delivery nanosystems for pharmaceutical applications. Antibiotics.

[12] Wang B., Chen S., Feng W., Shan X., Zhu X., Yuan R., Cao Y., Fan L., Yuan B., Wang H. (2024). Antimicrobial peptide-modified liquid metal nanomaterials for enhanced antibacterial photothermal therapy. Adv. Eng. Mater..

[13] Nie P., Zhao Y., Xu H. (2023). Synthesis, applications, toxicity and toxicity mechanisms of silver nanoparticles: A review. Ecotoxicol. Environ. Saf..

[14] Li Z., Song J., Yang H. (2023). Emerging low-dimensional black phosphorus: From physical-optical properties to biomedical applications. Sci. China Chem..

[15] Ouyang J., Feng C., Zhang X., Kong N., Tao W. (2021). Black phosphorus in biological applications: Evolutionary journey from monoelemental materials to composite materials. Acc. Mater. Res..

[16] Sun L., Han Y., Zhao Y., Cui J., Bi Z., Liao S., Ma Z., Lou F., Xiao C., Feng W. (2024). Black phosphorus, an advanced versatile nanoparticles of antitumor, antibacterial and bone regeneration for OS therapy. Front. Pharmacol..

[17] Zhang L., You J., Lv H., Liu M., Quni S., Liu X., Zhou Y. (2023). Black phosphorus—A rising star in the antibacterial materials. Int. J. Nanomed..

[18] Xu Y., Chen S., Zhang Y., Wu C., Li L., Hu X., Zhang J., Wang Y. (2023). Antibacterial black phosphorus nanosheets for biomedical applications. J. Mater. Chem. B.

[19] Guo T., Zhuang S., Qiu H., Guo Y., Wang L., Jin G., Lin W., Huang G., Yang H. (2020). Black phosphorus nanosheets for killing bacteria through nanoknife effect. Part. Part. Syst. Charact..

[20] Liang M., Zhang M., Yu S., Wu Q., Ma K., Chen Y., Liu X., Li C., Wang F. (2020). Silver-laden black phosphorus nanosheets for an efficient in vivo antimicrobial application. Small.

[21] Xu Y., Jiang X., Ge Y., Guo Z., Zeng Z., Xu Q., Zhang H., Yu X., Fan D. (2017). Size-dependent nonlinear optical properties of black phosphorus nanosheets and their applications in ultrafast photonics. J. Mater. Chem. C.

[22] Chen W., Ouyang J., Liu H., Chen M., Zeng K., Sheng J., Liu Z., Han Y., Wang L., Li J. (2017). Black phosphorus nanosheet-based drug delivery system for synergistic photodynamic/photothermal/chemotherapy of cancer. Adv. Mater..

[23] Liu Y., Tan Y., Cheng G., Ni Y., Xie A., Zhu X., Yin C., Zhang Y., Chen T. (2024). Customized intranasal hydrogel delivering methylene blue ameliorates cognitive dysfunction against Alzheimer’s disease. Adv. Mater..

[24] Zhang H., Sun C., Xu N., Liu W. (2024). The current landscape of the antimicrobial peptide melittin and its therapeutic potential. Front. Immunol..

[25] Liu S., Teng L., Ping J. (2024). Graphitic carbon nitride confers bacterial tolerance to antibiotics in wastewater relating to ATP depletion. Molecules.

[26] Liu S., Ji Y., Zhu H., Shi Z., Li M., Yu Q. (2023). Gallium-based metal–organic frameworks loaded with antimicrobial peptides for synergistic killing of drug-resistant bacteria. J. Mater. Chem. B.

[27] Yu D., Wang Y., Zhang J., Yu Q., Liu S., Li M. (2022). Synthesis of the ternary nanocomposites composed of zinc 2-methylimidazolate frameworks, lactoferrin and melittin for antifungal therapy. J. Mater. Sci..

[28] Liu W., Zhang Y., Zhang Y., Dong A. (2020). Black phosphorus nanosheets counteract bacteria without causing antibiotic resistance. Chem. Eur. J..

[29] Virgo E.P., Haidari H., Shaw Z.L., Huang L.Z.Y., Kennewell T.L., Smith L., Ahmed T., Bryant S.J., Howarth G.S., Walia S. (2023). Layered black phosphorus nanoflakes reduce bacterial burden and enhance healing of murine infected wounds. Adv. Therap..

[30] Javed A., Balhuizen M.D., Pannekoek A., Bikker F.J., Heesterbeek D.A.C., Haagsman H.P., Broere F., Weingarth M., Veldhuizen E.J.A. (2023). Effects of *Escherichia coli* LPS structure on antibacterial and anti-endotoxin activities of host defense peptides. Pharmaceuticals.

[31] Wang M., Li Z., Zhang Y., Li Y., Li N., Huang D., Xu B. (2021). Interaction with teichoic acids contributes to highly effective antibacterial activity of graphene oxide on Gram-positive bacteria. J. Hazard. Mater..

[32] Ren T., Wang Y., Yu Q., Li M. (2019). Synthesis of antimicrobial peptide-grafted graphene oxide nanosheets with high antimicrobial efficacy. Mater. Lett..

[33] Peng L., Wei H., Tian L., Xu J., Li M., Yu Q. (2021). Phospholipid/protein co-mediated assembly of Cu_2_O nanoparticles for specific inhibition of growth and biofilm formation of pathogenic fungi. Sci. China Mater..

[34] Ahmed W., Li S., Liang M., Kang Y., Liu X., Gao C. (2024). Multifunctional drug- and AuNRs-loaded ROS-responsive Selenium-containing polyurethane nanofibers for smart wound healing. ACS Biomater. Sci. Eng..

[35] Yu J.R., Varrey P., Liang B.J., Huang H.-C., Fisher J.P. (2021). Liposomal SDF-1 alpha delivery in nanocomposite hydrogels promotes macrophage phenotype changes and skin tissue regeneration. ACS Biomater. Sci. Eng..

[36] Andoy N.M.O., Jeon K., Kreis C.T., Sullan R.M.A. (2020). Multifunctional and stimuli-responsive polydopamine nanoparticle-based platform for targeted antimicrobial applications. Adv. Func. Mater..

