# Supplementary material for: Biocompatible Black Phosphorus Nanosheets-Antimicrobial Peptide Nanocomposites for Enhanced Anti-Infection Therapy
Source: Molecules. 2025 Feb 14;30(4):872. doi: 10.3390/molecules30040872 (PMC11857953; doi:10.3390/molecules30040872)
Supplement: Supplementary file 1 [file molecules-30-00872-s001.zip › molecules-3468193-supplementary.pdf]

Biocompatible black phosphorus nanosheets-antimicrobial peptide nanocomposites for enhanced anti-infection therapy

Shuo Liu [1,] *, Zhishang Shi[2], Lin Teng[1], Junlian Nie[1] and Libing Zhang[3]

[1] School of Energy and Chemical Engineering, Tianjin Renai College, Tianjin 301636, China
[2] College of Life Sciences, Nankai University, Tianjin 300071, China
[3] Tianjin Key Laboratory of Molecular Optoelectronic Sciences, Department of Chemistry, School of Science, Tianjin University, Tianjin 300072, China
* Correspondence: liushuo@nankai.edu.cn

Table S1 Diameter and zeta potential of BPs and BPs/Mel.

	BPs-2	BPs/Mel-2	BPs-7	BPs/Mel-7	BPs-10	BPs/Mel-10
Diameter (nm)	430.6	504.8	201.73	363.19	71.47	143.6
polydispersity index (PDI)	0.31	0.26	0.15	0.21	0.24	0.12
Zeta potential (mV)	-27.5	36.5	-37.9	11.7	-20	36

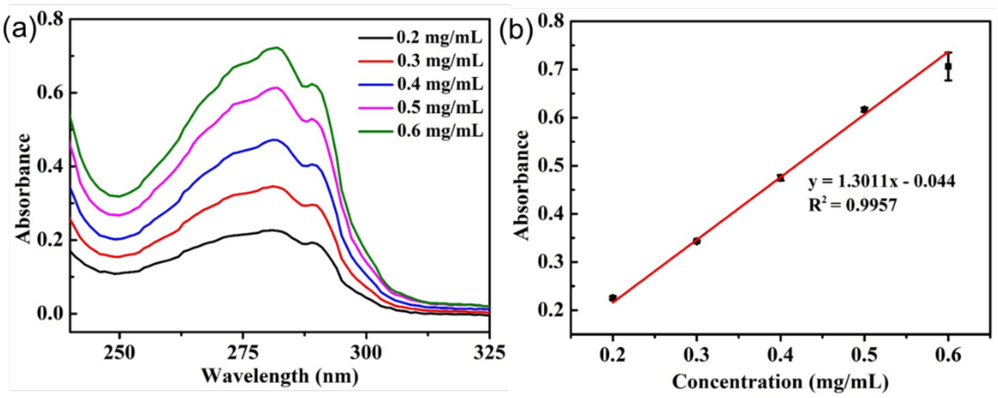

Figure S1 Calibration of Mel concentration in solution. (a) UV-Vis spectra of increasing concentrations of Mel in 10 mM PBS buffer (pH 7.4). (b) Calibration curve obtained from taking the absorbance at 282 nm in (a) and plotting them against the known concentrations of Mel. The curve was fitted with a linear function.

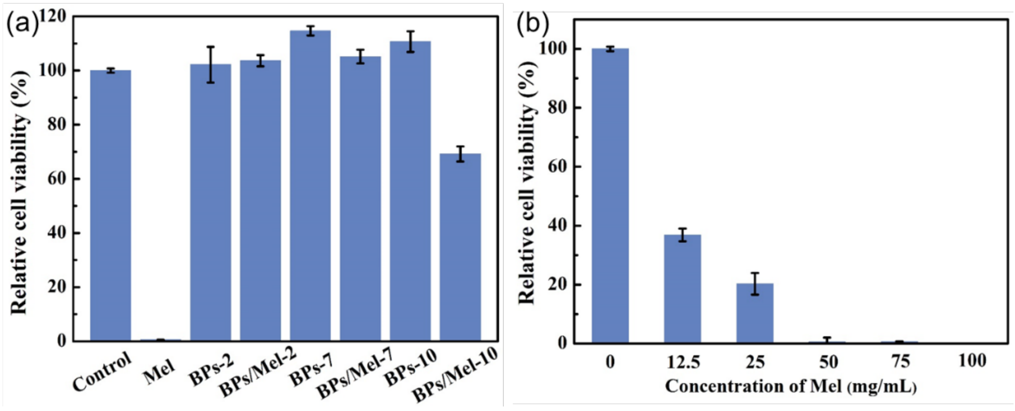

Figure S2 Cell cytotoxicity of Hela cells treated with Mel, BPs, and BPs/Mel. (a) Cell viability of Hela cells treated with 50 μg/mL Mel, BPs, and BPs/Mel. (b) Cell viability of Hela cells treated with Mel.
